# Embryonic development and egg viability of *w*Mel-infected *Aedes aegypti*

**DOI:** 10.1186/s13071-019-3474-z

**Published:** 2019-05-06

**Authors:** Luana Cristina Farnesi, Thiago Affonso Belinato, João Silveira Moledo Gesto, Ademir Jesus Martins, Rafaela Vieira Bruno, Luciano Andrade Moreira

**Affiliations:** 1 Laboratório de Biologia Molecular de Insetos, Instituto Oswaldo Cruz, Fiocruz, Rio de Janeiro, RJ Brazil; 2 Laboratório de Fisiologia e Controle de Artrópodes Vetores, Instituto Oswaldo Cruz, Fiocruz, Rio de Janeiro, RJ Brazil; 3 Mosquitos Vetores: Endossimbiontes e Interação Patógeno-Vetor, Instituto René Rachou, Fiocruz, Belo Horizonte, MG Brazil; 4 Instituto Nacional de Ciência e Tecnologia em Entomologia Molecular (INCT-EM)/CNPq, Rio de Janeiro, Brazil

**Keywords:** *Aedes aegypti*, *Wolbachia*, *w*Mel strain, Desiccation resistance, Embryogenesis, Egg viability

## Abstract

**Background:**

*Aedes aegypti* is a major disease vector in urban habitats, involved in the transmission of dengue, chikungunya and Zika. Despite innumerous attempts to contain disease outbreaks, there are neither efficient vaccines nor definite vector control methods nowadays. In recent years, an innovative strategy to control arboviruses, which exploits the endosymbiotic bacterium *Wolbachia pipientis*, emerged with great expectations. The success of the method depends on many aspects, including *Wolbachia*’s cytoplasmic incompatibility and pathogen interference phenotypes, as well as its effect on host fitness. In this work, we investigated the influence the *Wolbachia* strain *w*Mel exerts on embryo development and egg viability and speculate on its field release use.

**Methods:**

Wild-type (Br or Rockefeller) and *Wolbachia*-harboring specimens (*w*MelBr) were blood-fed and submitted to synchronous egg laying for embryo development assays. Samples were analyzed for morphological markers, developmental endpoint and egg resistance to desiccation (ERD). Quiescent egg viability over time was also assessed.

**Results:**

*w*MelBr samples completed embryogenesis 2–3 hours later than wild-type. This delay was also observed through the onset of both morphological and physiological markers, respectively by the moments of germband extension and ERD acquisition. Following the end of embryonic development, *w*MelBr eggs were slightly less resistant to desiccation and showed reduced viability levels, which rapidly decayed after 40 days into quiescence, from approximately 75% to virtually 0% in less than a month.

**Conclusions:**

Our data revealed that the *w*Mel strain of *Wolbachia* slightly delays embryogenesis and also affects egg quality, both through reduced viability and desiccation resistance. These findings suggest that, although embryonic fitness is somehow compromised by *w*Mel infection, an efficient host reproductive manipulation through cytoplasmic incompatibility seems sufficient to overcome these effects in nature and promote bacterial invasion, as shown by successful ongoing field implementation.

## Background

The mosquito *Aedes aegypti* (= *Stegomyia aegypti*) is a major disease vector in urban habitats, being able to host and transmit dengue (DENV), yellow fever (YFV), chikungunya (CHIKV) and Zika (ZIKV) viruses [1, 2]. While DENV is the most prevalent, with global estimates pointing to approximately 400 million infections annually [3], the other arboviruses are emerging in new territories and augmenting their range and impact. In Central and South America, for example, the introduction of CHIKV resulted in approximately one million suspected disease cases between 2013 and 2014 [4]. A similar impact on public health occurred in the Americas after the introduction of the ZIKV, presumably between May and December 2013 [5].

To date, vaccines or antiviral drugs for CHIKV and ZIKV are still not available and those for DENV serotypes have shown little efficacy and need further improvement [2, 6–8]. Therefore, the current strategies to reduce the transmission of these viruses are mostly aimed at suppressing mosquito populations. While mechanical control of breeding sites should not be disregarded, effectively mapping and accessing those sites, as well as properly engaging community members, are major obstacles. Likewise, chemical control has also shown limited efficacy, mainly due to the surge of genetic variants with resistance to traditionally employed compounds [9–11]. Hence, the development of new disease control strategies is a primary goal with urgent public health needs.

An innovative and promising method for arbovirus control, involving the bacterial endosymbiont *Wolbachia pipientis*, has been proposed and successfully tested in *Ae. aegypti* [12–15]. Even though most insects naturally harbour one or more strains of *Wolbachia* [16], this mosquito species does not [17]. Instead, *Ae. aegypti* was artificially transinfected with *Wolbachia* strains from the fruit-fly *Drosophila melanogaster*, generating stable and heritable lines which are currently being used in control programmes [12–15, 18, 19]. A striking feature of *Wolbachia* is its ability to manipulate host reproductive biology to increase rates of maternal transmission in a non-Mendelian fashion, promoting its own dispersal through native mosquito populations [20]. This is achieved by triggering a phenomenon called cytoplasmic incompatibility (CI), which leads to unviable progeny when infected males mate with uninfected females [19–21]. In addition to that, some *Wolbachia* strains inhibit pathogen replication and dissemination across mosquito tissues, effectively reducing the transmission of DENV, CHIKV and ZIKV [13, 22, 23]. Altogether, these features highly encourage the use of *Wolbachia* in methods aiming at arbovirus control.

Given the success of initial field trials [15], the mass release of *Ae. aegypti* infected with *Wolbachia* has been proposed by the ‘World Mosquito Program’ (WMP) (previously the ‘Eliminate Dengue’ project) and is now part of public health initiatives in 12 countries (https://www.worldmosquitoprogram.org/). The whole strategy is based on the gradual replacement of natural mosquito populations, susceptible to arboviruses, by *Wolbachia*-harboring refractory counterparts [15, 19, 21]. The efficacy with which the bacteria spreads and invades new localities largely depends on fitness-related aspects of mosquito hosts, which can be observed at both physiological and behavioural levels [21, 24, 25]. Some *Wolbachia* strains, like the most pathogenic *w*MelPop, elicit higher fitness costs, as expressed by a clear reduction in longevity, egg viability and reproductive potential [21, 26–28]. On the other hand, strains like *w*Mel interact with the host without affecting its fitness as much, with only subtle effects on life-history traits [21], thus being considered the preferred choice for field-release application [15, 19]. However, characterization studies of other traits, as well as new *Wolbachia* strains and host background interactions, are still ongoing and represent an important step towards the improvement of current methods [29]. Still not much explored in such studies are aspects concerning embryo development and its capacity to withstand dry environments (aka eggs resistance to desiccation, ERD). Interestingly, recent reports revealed a viability decay of quiescent *w*Mel-infected eggs [30, 31], suggesting that embryonic processes leading to desiccation resistance could also be altered. This effect, however, could be restricted to this particular host genetic background, derived from an Australian population, not being replicated in another. Hence, due to its fundamental importance to fitness, contributing to the maintenance and spread of natural populations [32, 33], these aspects need to be further investigated in different backgrounds and any *Wolbachia*-driven effect underlined.

In this work, we investigated a *w*Mel-infected Brazilian strain of *Ae. aegypti* during the course and after the end of embryonic development, focusing on traits such as the permeability barrier formation and quiescent egg viability. By comparing infected *vs* non-infected individuals, we could identify important physiological nuances and speculate on field release scenarios.

## Methods

### Mosquito strains

Assays were performed with a *w*Mel-infected strain, *w*MelBr, and two uninfected ones, Br and Rockefeller (hereafter referred to as “Rock”). *w*MelBr was obtained by repetitive backcrossing (9×) of the original *w*Mel Australian strain with the Br strain, which is derived from a native population of Rio de Janeiro (Brazil) [19]. Following the backcrossing, *w*MelBr was often checked for the presence of *Wolbachia* as part of our routine maintenance and quality control. Both cytoplasmic incompatibility and maternal transmission rates were virtually 100% [19]. Even so, some random *w*MelBr samples used in this work were also checked, and all turned out positive for *Wolbachia* (data not shown). In order to avoid issues related to inbreeding or genetic drift, both *w*MelBr and Br were refreshed with wild-caught males in every 5 generations. The samples used here were derived from the 19th generation, and therefore refreshed three times. We believe this procedure was sufficient to keep genetic background homogeneous between *w*Mel-infected and non-infected strains, so that differences arising from their comparison must be driven by the bacterium and not by other factors related to laboratory adaptation.While Br served as an experimental control, the other uninfected strain, Rock, was added to the analysis as a methodological control because of its broad use in diverse mosquito biology studies [34], including those on embryogenesis [35].

### Mosquito rearing

Specimens were reared in laboratory standard conditions [36]. Immature stages were maintained in plastic trays with 1 l of dechlorinated water and fed fish food (Tetramin® Tropical Tablets, Tetra, Spectrum Brands). Adult mosquitoes were kept at 26 ± 2 °C and 70 ± 10% relative humidity (RH), on a 10% sucrose solution *ad libitum*. For egg production, females were blood-fed for 20 min with human-donated blood using Hemotek membrane feeders (Hemotek Ltd). To reduce the risk of arbovirus contamination, blood samples were previously tested for the presence of DENV using the Dengue NS1 Ag Strip test (BioRad Laboratories, Hemel Hempstead, United Kingdom).

### Synchronous egg laying

Three to four days after blood-feeding, groups of gravid females were anaesthetized on ice for one minute and transferred to Petri dishes (8.5 cm diameter) internally covered with filter papers. Next, these papers were carefully wet with dechlorinated water and kept at 25 ± 1 °C for 1 h in the dark, after which females were removed [36].

### Embryonic development endpoint assay

Embryonic development completion of mosquito strains was assessed at 25 ± 1 °C as previously described [37]. Briefly, in each experiment, 50 gravid females from each strain were allowed to lay eggs, which were randomly assorted into three groups of 50 (i.e. three replicates; a total of 150 eggs). Two hours before the predicted hatching time (of Rock specimens) [34], eggs were immersed in 0.15% (w/v) yeast solution, and hatching was monitored every hour. Hatching rates (%) were calculated by assessing the number of first-instar larvae (L1) in the Petri dish, compared to the total amount of eggs. Data spanning from 70 to 80 h post-laying were normalized to reference viability indexes and fit in cumulative strain-specific non-linear time series. Reference viability indexes were obtained by the average hatch rate of three egg samples (50 eggs each) submitted to 24 h incubation following the embryogenesis endpoint. Embryogenesis endpoint was defined as the time required for 50% larval hatching [37]. At least three independent experiments were performed for each strain.

### Analysis of egg resistance desiccation (ERD) acquisition

ERD acquisition was assessed according to previous studies with minor adaptations [35, 36, 38]. At distinct embryogenesis time points, replicates of 40 to 50 synchronous eggs were placed on a Whatman No. 1 filter paper and air-dried for 15 min. Next, shrunken and intact eggs were counted using a stereomicroscope, and ERD evaluated. Results were obtained after three independent experiments, in which the strains were simultaneously tested.

### Analysis of embryo morphology

Synchronized eggs were fixed and clarified as described in Trpis [39]. The embryonic morphology of 30 synchronized eggs was checked at 12, 14 and 18 ‘hours after egg laying’ (HAE) (before, during and after the ERD acquisition, respectively), in the three strains. Embryonic stages were analyzed and identified with the use of a stereomicroscope (SteREO Discovery.V12, Carl Zeiss Microscopy GmbH, Jena, Germany) [35, 40–42].

### Quiescent egg viability

Quiescent egg viability was investigated during the course of 90 days. For each strain, 3 groups of 100 inseminated females (3–5 days-old) were fed human blood and individually transferred to Petri dishes, internally covered with water-dampened filter paper, so they could oviposit. Quiescent eggs were kept at 26 ± 1 °C and 80 ± 10% RH for various periods of time (24 h, one week, and then weekly until 90 days), after which they were submitted to viability assays. Experiments consisted of randomly selecting 30 eggs from each group of females and checking the hatching rate after complete immersion in 0.15% yeast solution for 24 h [37].

### Statistical analyses

All statistical analyses were done with GraphPad Prism 6 (Graphpad Software, Inc). Data were plotted as means (± SEM) of three to five independent experiments (replicates). Following non-linear regression of datasets, curves were compared using the F-test.

## Results

### Embryonic developmental time

**Fig. 1:**
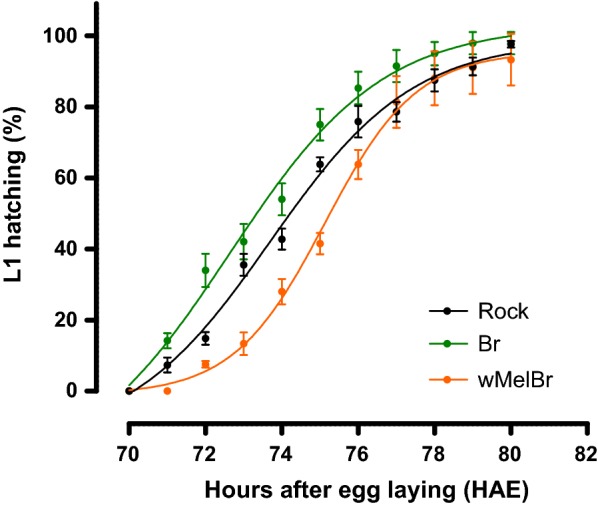
*Wolbachia w*Mel strain delays embryogenesis in *Aedes aegypti.* Following stimuli, cumulative larval hatching of wild-type (Br and Rock) and *Wolbachia*-infected (*w*MelBr) individuals was recorded from 70 to 80 hours after egg laying (HAE), yielding strain-specific non-linear time series, normalized by viability reference indexes (see “Methods” for details). Statistical comparisons point to significant differences between curves (F-test, *F*_(8,208)_ = 17.79, *P* < 0.0001), suggesting that *w*MelBr completes embryogenesis later in time. Data are represented by means (± SEM) of three independent experiments

To investigate the effect of *Wolbachia* on the developmental time of *Ae. aegypti* embryos, samples of *w*MelBr, Br and Rock (reference strain) were submitted to optimal hatching conditions and closely inspected till embryogenesis completion (i.e. 50% of larval hatching), as previously reported [37]. By scoring the embryonic developmental time as ‘hours after egg laying’ (HAE), *w*MelBr completed embryogenesis in 76.1 HAE, as opposed to 73.6 and 74.8 HAE in the Br and Rock controls, respectively (Fig. 1 and Table 1). Non-linear regression analysis further highlighted the strain differences with respect to their cumulative hatching curves (*F*_(8,208)_ = 17.79, *P* < 0.0001). Our data also revealed that, in addition to its prolonged developmental time, *w*MelBr embryos were less viable as compared to Br and Rock controls (Table 1).

**Table 1:** Embryonic development performance of wild-type and *w*Mel-infected *Ae. aegypti*

*Ae. aegypti* strain	Hours after egg laying			% Viability^c^
	Completion of embryogenesis^a^	First L1^b^	Last L1^b^	
*Rock*	74.8 ± 0.4	71	80	90.6 ± 3.2
*Br*	73.6 ± 0.5	71	79	96.3 ± 2.0
*w*MelBr	76.1 ± 0.4	72	80	75.3 ± 8.5

^a^Mean hatching time ± SD for 50% individuals

^b^L1, first-instar larvae

^c^Mean L1 hatching percentage ± SD following eclosion stimuli

### Egg resistance to desiccation (ERD) acquisition

**Fig. 2:**
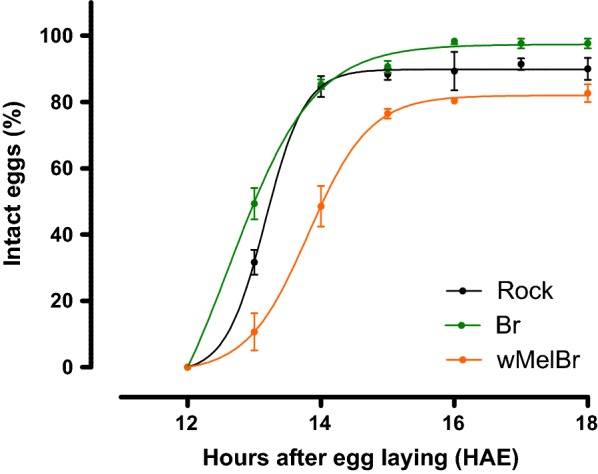
*Wolbachia w*Mel strain affects egg impermeability acquisition during embryonic development. Wild-type (Br) and Rockefeller (Rock) and *Wolbachia*-infected (*w*MelBr) eggs were air-dried at different times over embryonic development, and the percentage of intact ones (not shrunken) was registered. Non-linear regression analysis suggests that the presence of *w*Mel influences impermeabilization in developing eggs (a.k.a. egg resistance to desiccation phenotype) (F-test, *F*_(8,47)_ = 30.20, *P* < 0.0001). Data are shown by means (± SEM) of three independent experiments

**Fig. 3:**
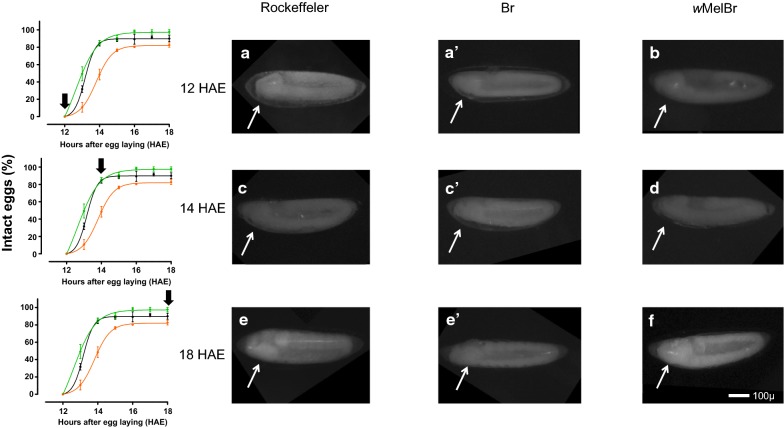
Comparative embryo morphology in *w*Mel-infected *vs* non-infected eggs: before, during and after impermeability acquisition. *Wolbachia*-infected (*w*MelBr) or non-infected eggs (Br and Rock) were clarified at 12, 14 and 18 hours after egg laying (HAE) and embryo morphology was analyzed under the microscope. **a**, **a′** 12-HAE embryo, at germ band extension. **b** 12-HAE embryo, at early germ band extension. **c**, **c′** 14-HAE embryo, at the maximum germ band extension. **d** 14-HAE embryo, at germ band extension. **e**, **e′** 18-HAE embryo, at the beginning of germ band retraction, showing embryo segmentation. **f** 18-HAE embryo, at maximum germ band extension, without strong segmentations. White arrows point to embryo heads, and black arrows refer to the moment of embryo collect and fixation to analysis. *Scale-bar*: 100 µm

To further investigate the developmental nuances of *Wolbachia*-harboring embryos, we assayed the egg resistance to desiccation (ERD) acquisition in each strain. For both controls (Br and Rock), ERD was acquired between 13 and 14 HAE, while for *w*MelBr this phenomenon occurred a few hours later, between 15 and 16 HAE (Fig. 2). Statistical comparisons of non-linear regression curves support these findings, pointing to significant differences in ERD profiles between *w*MelBr and the controls (*F*_(8,47)_ = 30.20, *P* < 0.0001). It is important to note that while ERD acquisition arises later in *Wolbachia*-harboring embryos, it seems that the relative time necessary to achieve 20% of complete embryogenesis is similar among all strains (compare Table 1 to Fig. 3).

### Embryo morphology during the ERD acquisition

Embryo images were obtained at 12, 14 and 18 HAE in all strains. As expected, no differences in embryo morphology were detected in both controls (Fig. 3, panels a-a′, b–b′ and e–e′). The maximum germ band extension occurred at 14 HAE in 70.1% and 72.7% of Rock and Br embryos, respectively (Table 2). On the other hand, *w*MelBr embryos showed a markedly delayed phenotype, with the maximum germ band extension occurring mostly (in approximately 80% of samples) at 18 HAE. At this point, non-infected embryos (Rock and Br) face the subsequent developmental stage called “germ band retraction” (Table 2).

**Table 2:** *Wolbachia w*Mel delays *Ae. aegypti* embryonic development checkpoints

	Rock	Br	*w*MelBr
Early germ band extension (12 HAE^a^)	93.6 ± 4.0	94.3 ± 5.1	11.7 ± 10.4
Maximum germ band extension (14 HAE^a^)	70.1 ± 1.2	72.7 ± 6.7	13.3 ± 13.2
Germ band retraction (18 HAE^a^)	97.0 ± 2.6	95.0 ± 18.6	18.3 ± 5.7

^a^HAE, hours after egg laying

*Note*: Values denote mean percentages ± SD of three independent experiments (see “Methods” for details)

### Quiescent egg viability

**Fig. 4:**
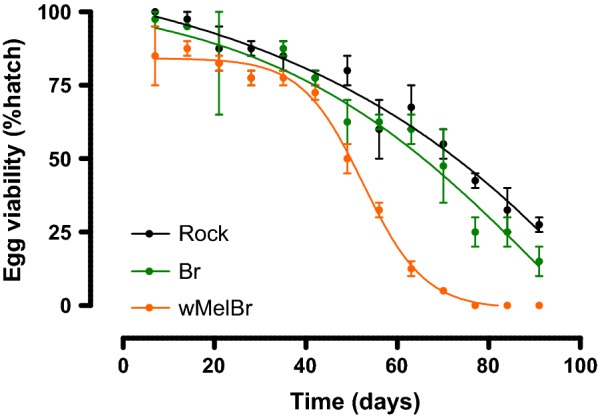
Quiescent egg viability of *Aedes aegypti* following *Wolbachia w*Mel infection. *Wolbachia*-infected (*w*MelBr) or non-infected (Br and Rock) quiescent eggs were tested for viability indexes (i.e. hatching percentage) over time. Our results revealed a significant decrease in viability due to *Wolbachia* infection, especially after 40 days (F-test, *F*_(8,66)_ = 27.92, *P* < 0.0001). Each point represents the mean (± SEM) of three independent experiments

Following ERD acquisition and storage in dry conditions, quiescent egg viability was investigated for three months at weekly intervals. As expected, all the three strains exhibited a decrease in viability over time. The decay pattern was very similar for both uninfected strains, Br and Rock, with a characteristic ‘quasilinear’ negative trend throughout time, but still revealing 60–70% viability indices after 60 days (Fig. 4). In contrast, the decay pattern for *w*MelBr was notably different, with a sudden drop in egg viability after 40 days till up to 80 days, when no larvae hatching was observed. Statistical comparisons between non-linear regressions curves corroborate these differences, suggesting that egg viability is significantly affected by the presence of *Wolbachia* (*F*_(8,66)_ = 27.92, *P* < 0.0001) but only after 40 days in dry conditions.

## Discussion

Vector control strategies are constantly evolving to fight deadly arbovirus epidemics, especially those affecting densely populated areas. In big cities, outbreaks of Zika, chikungunya and dengue are attributed to *Ae. aegypti*, a recurrent character in public health debates. This is partly due to its highly anthropophilic behavior and adaptability to urban habitats, as diverse as it could be [43, 44]. Females of this species use a large spectrum of artificial containers to lay their eggs, usually on the vessel wall just above the water column [33, 43]. Following the end of embryo development, the eggs may enter a quiescent state and become resistant to desiccation, withstanding dry periods without significantly losing viability [36, 45]. The ERD phenotype, acquired during early embryogenesis, is associated with the serous cuticle (SC) formation, an eggshell-like inner barrier constituted by an extracellular matrix of chitin that prevents the water outflow [35, 36, 38]. This feature gives the species the ability to keep local egg loads, bypassing months with low precipitation indexes and resuming development soon after environmental conditions allow. It may also help to explain the species passive dissemination around the globe, ‘hitch-hiking’ inside artificial containers of trading goods (e.g. used tires) [46]. Not surprisingly, the geographical spread of *Ae. aegypti* correlates to that of important arboviral diseases such as dengue [1, 3], confirming a causative relation between entities and reinforcing the need for targeting the vector effectively.

In the environment, *Ae. aegypti* females lay their eggs over a multitude of microhabitats, both natural and artificial, and usually on dark spots which are virtually impossible to fully assess [47, 48]. As a result, eggs are not much affected by current vector control methods based on breeding-site removal or chemical attack by pesticides [38]. In fact, these methods mostly target larvae and adults, leaving eggs intact for restoring population numbers following supposedly ‘successful’ suppression campaigns [49]. In the absence of methods to specifically target eggs, or even to suppress larvae and adults more efficiently and sustainably, alternative technologies based on population replacement have been developed and gained momentum in the last decade [13, 15, 19]. One innovative strategy uses the bacterium *W. pipientis* [12–15]. Through a mechanism involving the upregulation of basal immune system and disrupted cholesterol homeostasis [21, 24, 50], some strains of the bacterium interfere with pathogen replication inside the mosquito host, rendering it less able to transmit a wide variety of viruses [13, 23]. The level of interference is variable among strains [30, 31], and has been inferred for every host background-*Wolbachia* association before field application. *w*Mel, the most commonly used strain for population replacement strategies, has already been tested and exhibited high levels of refractoriness against dengue, Zika, chikungunya and Mayaro viruses, in diverse genetic backgrounds [13, 23, 30, 31, 51–53].

The successful invasion of *Wolbachia* depends on how fit infected individuals are in all life-cycle stages, being able to survive and mate in environmentally challenging conditions. Several studies have addressed the fitness of *w*Mel-infected hosts, revealing weak to mild costs on egg viability and longevity [14, 18, 19, 30, 31]. However, none of these studies has included the key embryogenesis traits such as the impermeability barrier formation (i.e. the ERD phenotype), despite its importance for vector population maintenance and spread to new territories.

In this work, we investigated the effects of the *w*Mel strain of *Wolbachia* on the embryonic development and viability of *Ae. aegypti* eggs from Brazil. Our results revealed that *w*Mel-infected individuals complete embryogenesis in approximately 76 HAE, which are a few hours later then non-infected controls (Fig. 1). This time delay could also be noticed in our assays to assess the ERD phenotype, first by measuring the percentage of intact eggs after forced air drying (Fig. 2), and second by monitoring the maximum germ band extension in embryo morphology (Fig. 3). Lastly, once ERD is expressed and embryogenesis is completed, we evaluated the viability of *w*Mel-infected quiescent eggs over time. During the first 40 days, our data revealed an expected decay in viability, with minimal difference between infected and non-infected strains. After 40 days, nonetheless, distinct profiles arose with a sudden drop in *w*MelBr viability indexes, reaching virtually basal levels at 60 days (Fig. 4). All these biologically relevant effects need to be understood from a fitness perspective and will be discussed hereafter.

Once *Wolbachia*-harboring mosquitoes are released and start to reproduce in the natural habitat, a broad and fierce competition for resources with native individuals shall take place. At the immature stage, competition for food inside the breeding sites is critical, especially in cases where there is low availability [54]. One could hypothesize that wild specimens, with a shorter embryonic developmental time, would hatch faster and have more immediate access to nutritional resources of the breeding site, whereas *Wolbachia*-harboring ones, which require a few more hours to complete development and hatch, would have a late access to the same resources. This effect, however, should be restricted to embryos skipping quiescence and hatching immediately after embryogenesis completion, otherwise a ‘time’ advantage for the wild-type would not be sustained. In any case, whether this is detrimental for *Wolbachia* in nature, and at what degree, is still unknown and needs to be assessed in future studies. Probably, a more complex environment *versus* host interaction, with daily and seasonal variations, would likely affect *Wolbachia* titers and modulate its influence on embryogenesis, alleviating or further enhancing the longer developmental time [29, 55–57]. Environmental heat stress, for instance, appears to reduce *w*Mel titers along with egg viability, maternal transmission and CI [57]. In spite of these putative consequences, it is important to highlight that developmental fitness must include traits other than the time required for embryogenesis completion, such as all the variables affecting the larvae until adulthood. As such, a simple process like speeding up the transition between larval stages, through increased feeding and metabolic rates, could compensate a longer embryogenesis time. Supporting this view, it has been demonstrated that *w*Mel-infected larvae develop faster than the wild-type in higher larval densities, keeping energetic reserves stable (i.e. glycogen levels) [58].

Although embryogenesis was slightly delayed in *w*Mel-infected individuals, key processes were preserved. The mere manifestation of ERD phenotype suggests that *Wolbachia* does not prevent metabolic pathways leading to cuticle deposition related to waterproofing. There seems to be, however, a small interference by the bacterium in this phenotype, as levels of intact eggs following a drying treatment appear to be lower in *w*Mel-infected samples (Fig. 2). Future studies shall address if this effect represents any fitness cost in the natural habitat.

Our data also revealed that, despite being less resistant to desiccation, *w*Mel-harboring eggs are able to maintain high viability rates (~ 75%) till approximately 40 days under quiescence, after which rates suffer a marked drop (Fig. 4). In an alternative host background (Australian), quiescent viability of *w*Mel-infected eggs also decays faster than non-infected controls, following a consistent negative trend, though not exhibiting a marked drop until week 10 [30, 31]. A difference observed here is probably due to interactions with the host genetic background, which puts fitness evaluation of under a local- or population-specific perspective. Nonetheless, at least for the first 40 days (or about 6 weeks), quiescent viability indexes were quite similar (i.e. 70–80%) in both Brazilian and Australian backgrounds. We believe that, for most places, this period is sufficient to allow the invasion of *Wolbachia* into wild populations, considering the successful establishment of *w*Mel in Australia following three years after field deployment [18, 59], and more recently in Brazil [60]. An exception may be some places with long standing dry seasons. In India, for instance, if *Wolbachia* fails to invade local populations during the rainy season, then the natural egg storage might not withstand in the wild long enough and remain viable until the next rain, ruining the previous program efforts. However, should *Wolbachia* benefit from the rainy season and successfully invades in the short run, the dry season would mean a suppression valve, crashing vector populations afterwards. This collateral effect, although not formally planned for replacement strategies, could be a desired consequence for some control campaigns, as it was suggested by previous studies [61].

Finally, this work provided original and compelling evidence on the effect elicited by the *w*Mel strain of *Wolbachia* on embryo development and egg viability. Importantly, these effects could possibly be restricted to the *w*Mel strain and its association with a Brazilian genetic background [19, 21, 60], thus one cannot extrapolate them without further testing. For this reason, we believe that the characterization of new *Wolbachia* strain/host interactions are worth the effort towards more flexible solutions, adapting the strategy to environmentally distinct locations [29, 57]. In this regard, we encourage a thorough evaluation of fitness aspects, including those related to development, of *Wolbachia*-harboring samples before and after field release. Meanwhile, we support the view that *w*Mel is a suitable choice for controlling arbovirus transmission in places often stricken by serious outbreaks of dengue, Zika and chikungunya. Considering its adaptive value in the field, the mere ERD expression shall provide *w*Mel-harboring lines with fundamental ‘embryonic’ fitness, contributing to its spread and perpetuation in most natural habitats. Only the continuous surveillance of vector population and *Wolbachia* prevalence before, during and after release efforts will gather information on how efficient the strategy is in the real world, helping scientists and public health agents to decide which improvements are needed.

## Conclusions

Our results revealed that the *w*Mel strain of *Wolbachia* elicit a small delay on host embryogenesis, also interfering with but not preventing the expression of the so-called ERD phenotype. Egg viability was not much affect by the bacterium following a short-term storage, yet the same is not true for periods over 40 days, when a critical decay in levels was observed. Altogether, these data contribute to new fitness evidence for the *w*Mel strain and acknowledge its importance in diverse field release scenarios. Through a strong self-driven component, *w*Mel seems to be able to fight weak to mild fitness costs and invade wild populations, thus being the current strain of choice in Brazil and other countries contemplated by the ‘World Mosquito Program’.

## Abbreviations

ERD: egg resistance to desiccation
HAE: hours after egg laying
